# Best practices for analyzing imputed genotypes from low-pass sequencing in dogs

**DOI:** 10.1007/s00335-021-09914-z

**Published:** 2021-09-08

**Authors:** Reuben M. Buckley, Alex C. Harris, Guo-Dong Wang, D. Thad Whitaker, Ya-Ping Zhang, Elaine A. Ostrander

**Affiliations:** 1 Cancer Genetics and Comparative Genomics Branch, National Human Genome Research Institute, National Institutes of Health, 50 South Drive, Building 50, Room 5351, Bethesda, MD 20892 USA; 2 State Key Laboratory of Genetic Resources and Evolution, Kunming Institute of Zoology, Chinese Academy of Sciences, Kunming, 650223 China; 3 Center for Excellence in Animal Evolution and Genetics, Chinese Academy of Sciences, Kunming, 650223 China

## Abstract

Although DNA array-based approaches for genome-wide association studies (GWAS) permit the collection of thousands of low-cost genotypes, it is often at the expense of resolution and completeness, as SNP chip technologies are ultimately limited by SNPs chosen during array development. An alternative low-cost approach is low-pass whole genome sequencing (WGS) followed by imputation. Rather than relying on high levels of genotype confidence at a set of select loci, low-pass WGS and imputation rely on the combined information from millions of randomly sampled low-confidence genotypes. To investigate low-pass WGS and imputation in the dog, we assessed accuracy and performance by downsampling 97 high-coverage (> 15×) WGS datasets from 51 different breeds to approximately 1× coverage, simulating low-pass WGS. Using a reference panel of 676 dogs from 91 breeds, genotypes were imputed from the downsampled data and compared to a truth set of genotypes generated from high-coverage WGS. Using our truth set, we optimized a variant quality filtering strategy that retained approximately 80% of 14 M imputed sites and lowered the imputation error rate from 3.0% to 1.5%. Seven million sites remained with a MAF > 5% and an average imputation quality score of 0.95. Finally, we simulated the impact of imputation errors on outcomes for case–control GWAS, where small effect sizes were most impacted and medium-to-large effect sizes were minorly impacted. These analyses provide best practice guidelines for study design and data post-processing of low-pass WGS-imputed genotypes in dogs.

**Supplementary Information:**

The online version contains supplementary material available at 10.1007/s00335-021-09914-z.

## Introduction

The price per marker for a genotyping assay can have a large influence on the success of genetic association studies. In dogs, DNA genotyping arrays, which provide hundreds of thousands of genotypes at relatively low costs, are highly beneficial for mapping loci (Awano et al. 2009; Hayward et al. 2016), characterizing genetic architecture (Boyko et al. 2010; Friedrich et al. 2019), and defining breed and population structure (Ali et al. 2020; Shannon et al. 2015). However, DNA genotyping arrays are limited by various known and unknown biases that occur during marker selection and probe design that cannot be removed without redesigning a new DNA array, which is an expensive and time-consuming process. An alternative similarly priced approach is low-pass whole genome sequencing (WGS) and imputation (Martin et al. 2021). Rather than assigning genotypes based on high confidence calls across a finite set of loci, low-pass WGS combines information from millions of randomly sampled low-confidence variant calls to impute likely genotypes from a reference panel, comprised of a large collection of WGS datasets representing potential haplotypes found within a population. Since low-pass WGS isn’t biased toward sampling specific loci, a major limiting factor is the reference panel used. Therefore, the utility of previous datasets can only improve with updated reference panels and is not hampered by acquisition bias of predetermined sites.

Due to its flexibility and scalability, low-pass sequencing and imputation has been applied to humans (Rubinacci et al. 2021; Wasik et al. 2021) and other mammalian species (Benjelloun et al. 2019; Nosková et al. 2021; Piras et al. 2020; Snelling et al. 2020). Results in humans demonstrate that low-pass WGS and imputation provide more accurate genotypes than those imputed using array data, leading to increased power for genome-wide association studies (GWAS) and more accurate polygenic risk score calculation. Piras et al. (2020) used low-pass WGS and imputation to identify candidate loci for canine idiopathic pulmonary fibrosis in West Highland white terriers (CPSF7 and SDHAF2). While successful in this case, the existing 350 dog breeds present a unique problem for conducting GWAS studies, as the existing structure of each breed, its history, and genome homogeneity are distinct (Ostrander et al. 2017). In the absence of empirical evidence for developing optimal strategies for study design and data processing, the probability of poor performance and misleading results is unknown. As many dog breeds and populations have only been sequenced to low levels, the development of a generalizable set of rules for low-pass WGS imputation across breeds would convert much of the existing data from low to high applicability, thus accelerating the dog as a genetic system for studies of canine and human health.

Here, we present an analysis of imputation accuracy of low-pass WGS in the context of canine genomics and establish optimized approaches for study design and data processing. We analyzed imputed genotypes from 97 test samples from 58 different breeds, many of which are not included in the reference panel containing the haplotypes that were used for imputation. We assessed the impact of minor allele frequency (MAF) on genotyping accuracy and determined whether it was better to use either MAFs generated from imputed genotypes or MAFs used from reference population genotypes for filtering purposes. Finally, we investigate the impact of imputation errors on study design by determining the necessary sample sizes and case–control ratios for a sufficiently powered case–control GWAS.

## Results

### Breed composition of the imputation reference panel and test datasets

**Fig. 1:**
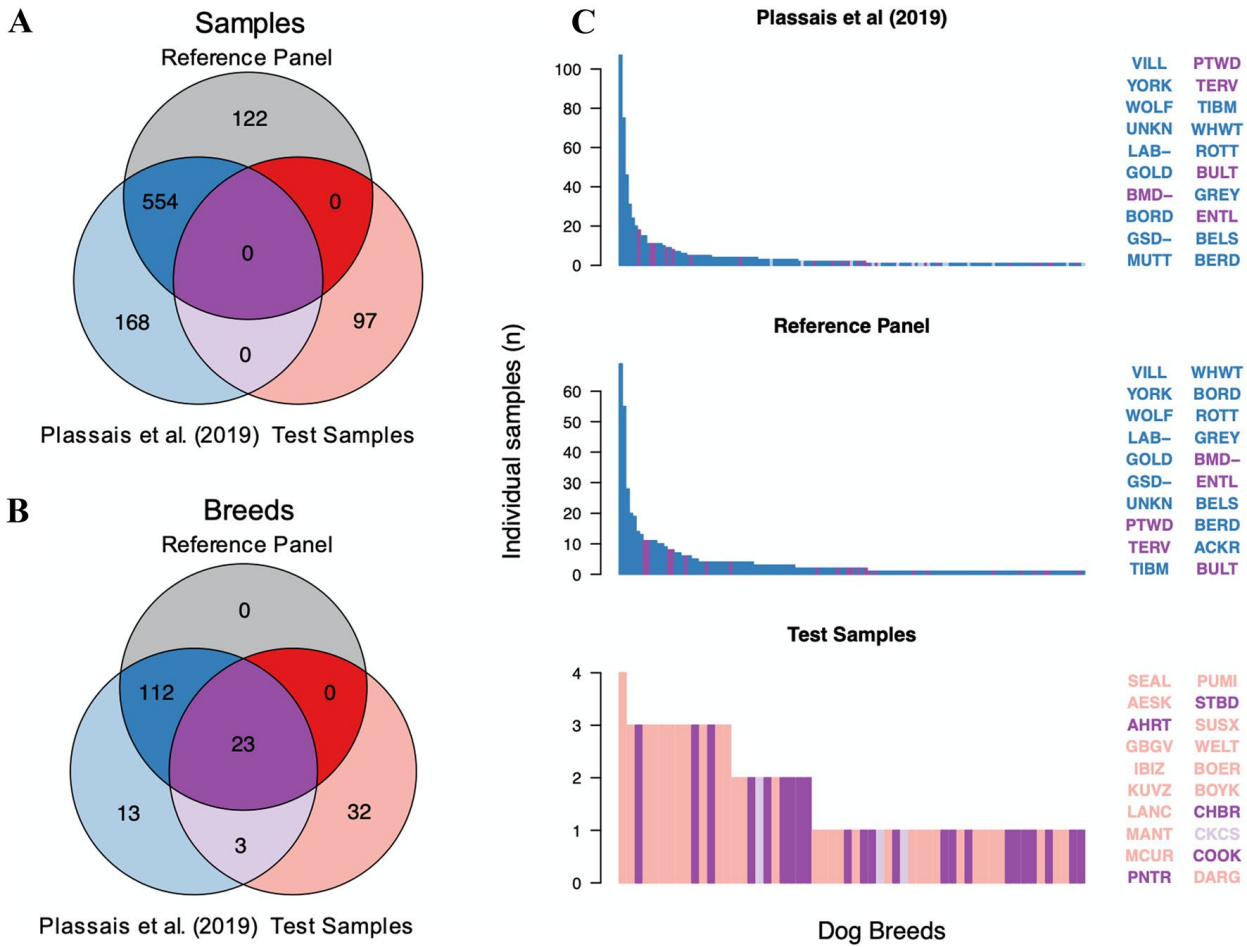
Test samples belong to a wide variety of breeds with most breeds likely not found within the imputation reference panel. **a** Sample membership within each dataset. Reference panel IDs could not always be linked to a publicly available dataset. **b** Breed membership among each dataset. Reference panel dogs whose IDs could not be linked to a publicly available sample have no breed label. **c** Breed frequency across each dataset. Using the colors from the Venn diagram in B, bar colors represent the population a specific breed can be found in. Labels to the left of each bar chart identify the 20 most common breeds. Breeds in bar charts are sorted by most to least common

The test dataset used for assessing imputation accuracy consists of 97 samples that were sequenced as part of the initial release of the Dog10K consortium. The test samples met the selection criteria outlined in the methods (Ostrander et al. 2019). The imputation reference panel used by Gencove, Inc. (New York, NY) consisted of 676 samples. Although the reference panel genotypes were not available for our analysis, the IDs of each individual sample were provided. Of these, 554 of 676 were matched to a previously published dataset, referred to as the Plassais et al. (2019) dataset (Fig. 1a) (Supplementary Table S1). Since a large portion of samples are shared between the Gencove reference panel and Plassais et al. (2019), we opted to use the publicly available Plassais et al. (2019) VCF file as a stand-in for the reference panel VCF, allowing us to estimate the variant allele frequencies of the imputation reference panel used by Gencove. The individual breeds in the Gencove reference panel samples were compared to the breeds in the Plassais et al. (2019) dataset and breeds from our test dataset (Fig. 1b). Only 13 breeds were identified as unique to the published Plassais et al. (2019) dataset and they ranged from one to three members each (Fig. 1c) (Supplementary Table S2). The remaining breeds were shared between the Gencove reference panel and the Plassais et al. (2019) dataset and typically contained similar numbers of individuals, with village dogs (VILL), Yorkshire terriers (YORK), wolves (WOLF), Labradors (LAB-), golden retrievers (GOLD), and unknown breeds (UNKN) being the six most popular breed designations in both datasets (Fig. 1c). Within the test samples, 23 breeds were shared with the reference panel and four breeds were shared with the Plassais et al. (2019) dataset only, while 32 breeds were unique to the test samples (Fig. 1b). In terms of member frequency per breed, the test samples had no more than four Sealyham terriers, the most common breed within the test samples. In addition, the total members per breed were relatively evenly distributed between breeds unique to the test samples and breeds found in other datasets (Fig. 1c).

To evaluate breed representation at a higher level, we determined the clade membership of all known breeds across the test dataset and the reference panel dataset using approaches described previously (Methods) (Table 1) (Supplementary Table S3). Of the 23 established domestic dog clades, only the Pinscher and Hungarian clades were not represented in the reference panel. This is, perhaps, because each of these clades contains only two breeds (Parker et al. 2017). Other clades of concern due to underrepresentation include the American terrier, Asian toy, small spitz, and toy spitz clades, which have less than three representative samples within the known breeds of the reference panel. Finally, there were 31 dogs from 14 breeds within our test samples with no previously assigned clade (Supplementary Table S4). Since most of these breeds are of European origin, a population of breeds highly represented in many analyses, they would likely be representative of previously identified clades. Together, this data suggest that the Plassais et al. (2019) dataset likely represents most of the same haplotypes found in the reference panel used for imputation, and that the test samples represent a mixture of both reference panel and unique breeds. The variation of reference breed representation is appropriate for defining a representative range of imputation accuracies for low-pass sequencing.

**Table 1:** Clade representation of reference and test datasets

Clades	Parker et al. (2017)^**a**^		Plassais et al. (2019)^**b**^		Test Samples	
	Samples	Breeds	Samples	Breeds	Samples	Breeds
Alpine	26	3	20	4	5	3
American Terrier	16	3	1	1	4	2
American Toy	20	2	5	2	0	0
Asian Spitz	83	9	25	9	1	1
Asian Toy	44	5	2	2	2	2
Continental Herder	44	5	25	4	2	2
Drover	34	4	15	4	0	0
European Mastiff	139	16	24	11	7	5
Hungarian	9	2	0	0	3	1
Mediterranean	98	14	12	6	7	3
New World	45	7	24	7	3	2
Nordic Spitz	64	5	13	10	7	4
Pinscher	12	2	0	0	1	1
Pointer Setter	88	12	18	10	5	3
Poodle	72	8	20	6	3	2
Retriever	66	7	48	7	3	2
Scent Hound	71	8	14	6	1	1
Schnauzer	20	2	5	2	0	0
Small Spitz	14	2	1	1	1	1
Spaniel	44	5	14	5	2	1
Terrier	140	18	100	15	7	6
Toy Spitz	32	4	2	2	2	2
UK Rural	145	16	42	12	0	0
Unplaced*^c^	20	2	7	4	0	0
Unknown breed*^d^	0	–	13	–	0	–
Village Dogs*^e^	0	–	69	–	0	–
Mix Breed*^f^	0	–	6	–	0	–
Wild Canids*^g^	9	2	29	2	0	0
No Clade Info*^h^	0	0	0	0	31	14

*Groups of samples that either do not form a monophyletic clade or have not been included in previous phylogenetic analyses

^a^Analysis that initially defined breed clade membership

^b^Dogs in the reference panel that are also included in Plassais et al. (2019)

^c^Dog breeds that formed their own branch in previous phylogenetic analysis and were not a member of a clade

^d^Dogs with no corresponding breed information

^e^Non-breed dogs sampled from 14 distinct geographic regions

^f^Dogs with mixed breed ancestry

^g^Group consists of gray wolves and golden jackals and breed label is used to differentiate these two different species

^h^Breeds not included in previous phylogenetic analyses and, therefore, not assigned clade membership

### Downsampling and imputation

The 97 test samples were each downsampled to a coverage level of 1× and underwent imputation using loimpute as part of the Gencove, Inc. platform (Wasik et al. 2021). The average read coverage of WGS variants and imputed variants was 17.5× and 1.06×, respectively (Supplementary Table S5). A single sample, Pointer06, had a mean coverage of 1.67x, an outlier compared to next highest coverage dog which was 1.30×, suggesting Pointer06’s original coverage levels were incorrectly estimated. However, since variation in coverage level is a potential outcome of low-pass sequencing, Pointer06 was retained for further analyses. Imputation returned 53,649,170 million (M) variant sites, consisting of 35,875,925 SNV and 17,773,245 indel sites. Most sites were homozygous for the reference allele across all samples and were, therefore. removed from the analysis, leaving a total of 14,845,499 SNVs and 7,946,973 indels. Alternatively, genotype calling of high-coverage WGS data for the test samples resulted in 18,476,517 SNVs and 12,831,692 indels.

**Fig. 2:**
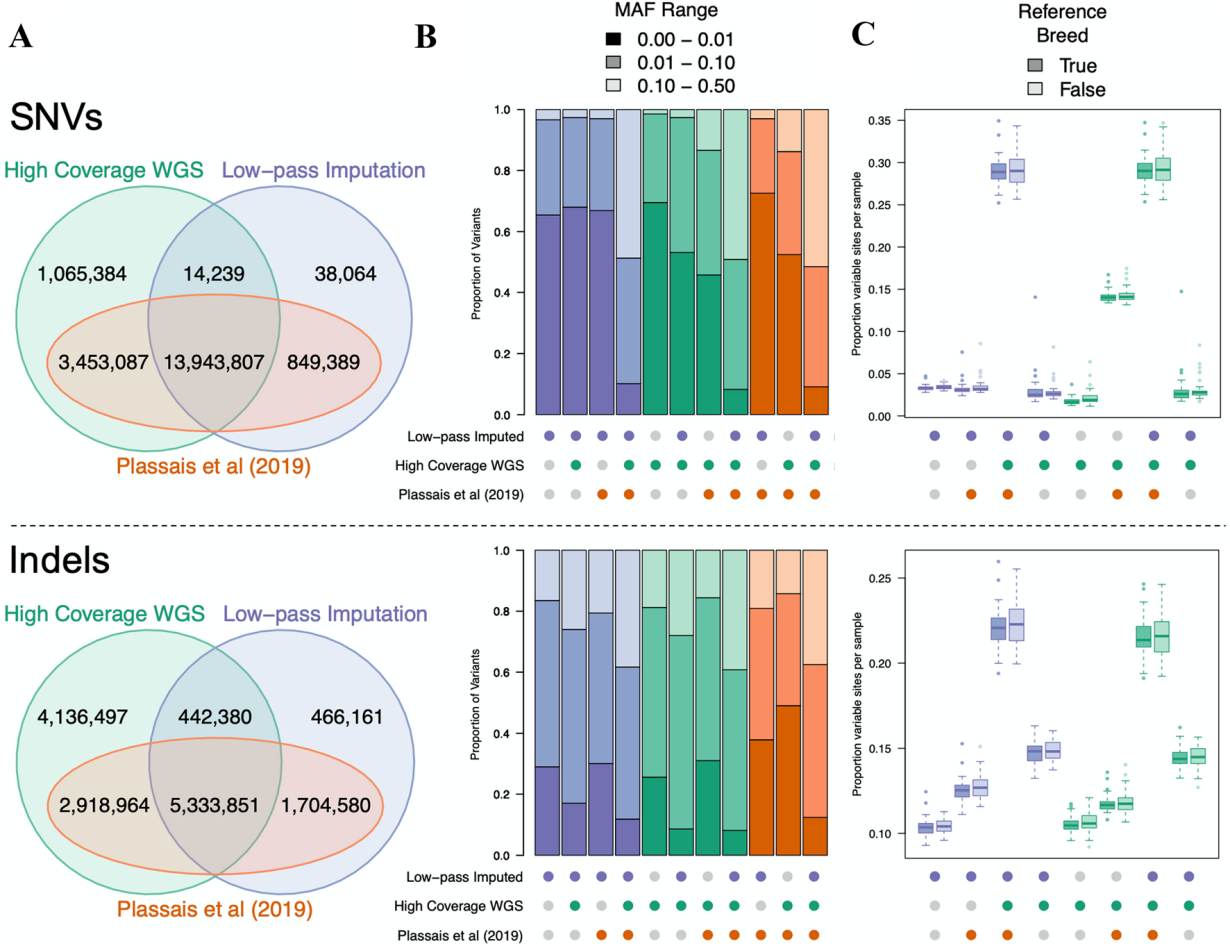
Genomic variant positions and their corresponding alleles are consistent across datasets. **a** Venn diagrams for SNVs and indels showing variants unique and shared across datasets. Datasets include the high-coverage WGS variant sites and low-pass imputed variant sites found across the 97 test samples and variants discovered in Plassais et al. (2019). Variants were identified as shared across datasets if the variant position, reference allele, and alternate allele were identical. **b** MAF distribution of each variant group from A. Variant groups are indicated by colored circles beneath the bar chart. Groups contain variants which are the intersect between the colored circles and do not contain variants found in the datasets represented by the gray circles. The color of each bar indicates the dataset used to calculate the MAF distribution and the shading level indicates the relevant MAF range. **c** Sites per sample in each variant group, where variant groups are presented as in B. Sites per sample are measured as the proportion of total sites within the relevant variant group that contain a non-reference allele for a particular sample. Samples have also been divided into two groups based on whether the respective breed also belongs to the Plassais et al. (2019) dataset and is, therefore, likely used in the imputation reference panel

To analyze the accuracy of imputation, we identified variants shared between the following groups: low-pass imputation, high-coverage WGS, and the Plassais et al. (2019) dataset. The majority of SNVs, 13,943,807, were shared between all three variant groups, which represented 93.9% of all imputed SNVs. Similarly, the majority of indels, 5,333,851, were also shared between the three variant groups. However, shared indels represented only 67.1% of all imputed indels, a smaller fraction than observed with SNVs. Importantly, 99.6% of all imputed SNV sites and 88.6% of indel sites were present within the Plassais et al. (2019) dataset, indicating its utility as a stand-in for the reference panel (Fig. 2a).

Although there were high levels of agreement between the low-pass imputation and the high-coverage WGS variant groups, many sites were specific to only one variant group. Since these sites were removed from our downstream analysis, we measured their MAF distributions to determine their potential impact on imputation accuracy. Variant group specific sites usually had MAFs < 0.01, whereas variants that were shared across all groups typically had higher MAFs (Fig. 2b). This indicates that high-coverage WGS-specific sites are likely singletons and absent from the imputation reference panel. Although 3.5 M of these sites are also found in the Plassais et al. (2019) dataset, they may belong to test sample breeds that were not present in the imputation reference panel. Conversely, low-pass imputation-specific sites are instead due to imputation errors. These sites are imputed as variable, when, according to the high-coverage data, they are homozygous for the reference allele. Since these low-pass imputation-specific sites tend to present as rare variants (MAF < 0.01), they are easily filtered out by MAF cutoffs typically used in association analyses and, thus, will only have a minor impact on any future analysis.

To determine whether breed composition of the reference panel has an impact on variant imputation within out test samples, we counted the number of variant sites per individual for each combination of the variant groups and compared the results between reference panel breeds and non-reference panel breeds. Results show that reference panel breeds and non-reference panel breeds carry a similar number of variant sites for both SNVs and indels, indicating that the breed composition of the current reference panel has little impact on variant detection from imputation (Fig. 2c). Ultimately, the total number of variant sites per samples varied according to MAF distributions, as shown in Fig. 2b.

### Filtering strategies to optimize accuracy

**Fig. 3:**
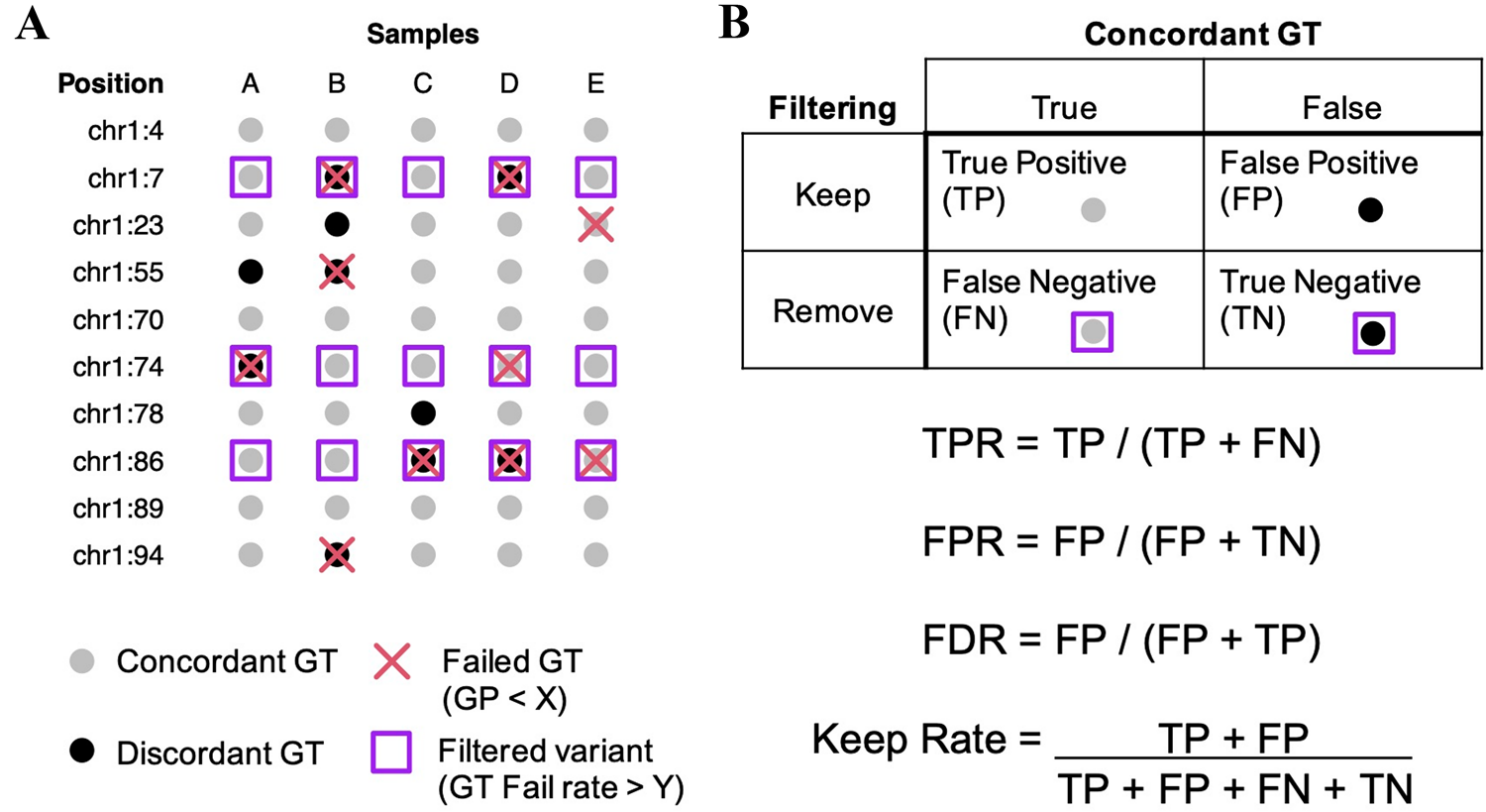
Filtering strategies for reducing imputation errors. **a** Schematic of imputed genotypes. Genotypes are represented as filled in circles, where black circles indicate discordant genotypes and gray circles indicate concordant genotypes. In this example, the genotypes themselves, such as heterozygous and homozygous, are hidden as they are not relevant. Generally, genotype concordance between actual and imputed data remains unknown and other alternative metrics are used to filter out sites that likely contain an abundance of imputation errors. Here, max genotyping probability (GP) is used to assess genotyping confidence. GP below a certain threshold, X, identifies low-confidence genotypes, which are marked with a red cross. Genomic positions that contain greater than a certain number of low-confidence genotypes are filtered out as their low-confidence genotyping rate is above the threshold Y. Here, sites with a low-confidence genotyping rate > 20%, or 1 out of 5 samples, are marked with purple squares. Ideally, sites removed by filtering are enriched for discordant genotypes. **b** The statistics are used to assess and compare filtering strategies. These include, true-positive rare (TPR), false-positive rate (FPR), false discovery rate (FDR), and keep rate, which is measured as the proportion of genotypes remaining after filtering

**Fig. 4:**
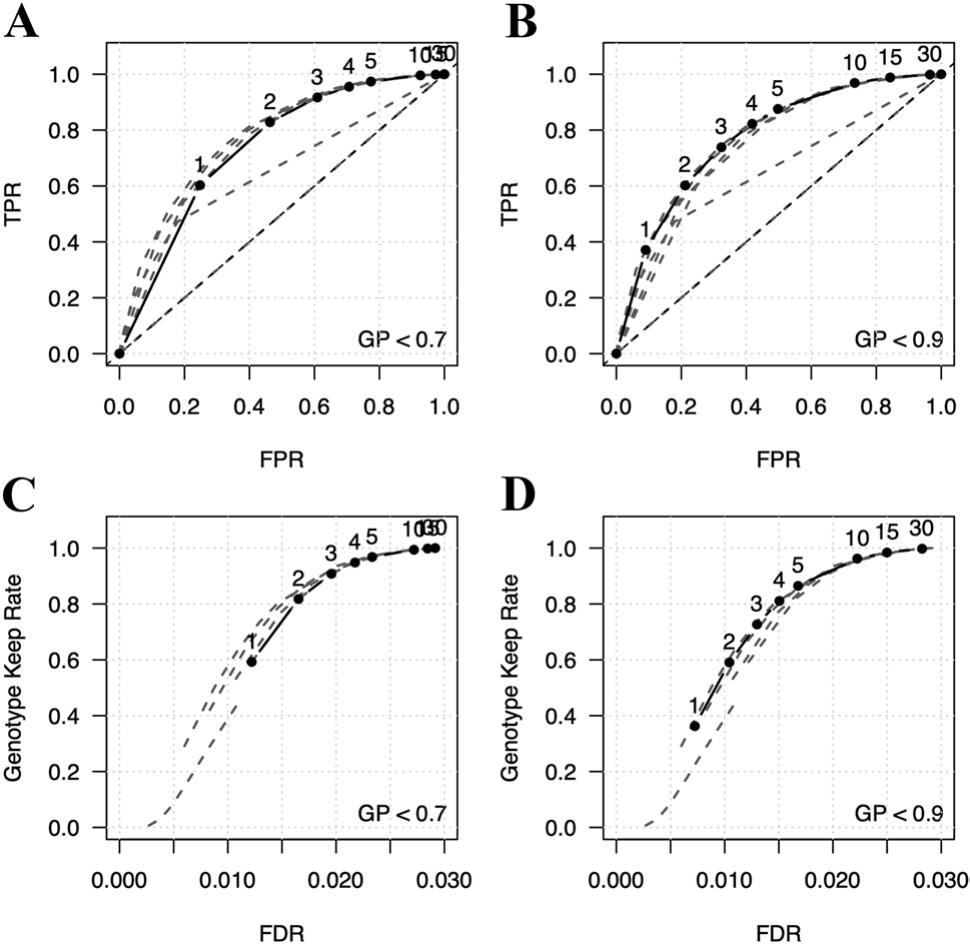
Performance of filtering strategies for reducing imputation errors. **a** ROC curve, where genotypes with GP < 0.7 are identified as low confidence (solid line). Numbers above each point along the solid line represent low-confidence rate thresholds for removing sites. These values and their ordering are identical across all four panels. Sites with a total number of low-confidence genotypes greater than or equal to the threshold are removed. Gray dashed lines represent ROC curves for other confidence threshold values. **b** ROC curve for confidence threshold set at GP < 0.9. **c** The proportion of variants remaining after filtering genotypes at GP < 0.7 and the corresponding FDR. As in C, the numbers above each point represent low-confidence rate threshold values and gray dashed lines represent curves for other confidence thresholds. **d** Proportion of variants remaining and their corresponding FDR after filtering at GP < 0.9

Filtering strategies are optimized by analyzing the relationships between imputation accuracy, genotyping confidence as determined by max genotype probability (GP), and low-confidence genotype threshold (Methods) (Fig. 3a). Here, genotype probability is defined as the posterior probability produced by the imputation algorithm implemented in the loimpute software tool and low-confidence genotype threshold is defined as the allowable number of low-confidence genotypes for a site to contain (Wasik et al. 2021). To evaluate optimal filtering strategies across the range of GP values and low-confidence genotyping rates, we compared the number of correctly imputed genotypes to the number of incorrectly imputed genotypes in several different ways (below) (Fig. 3b). Receiver operator characteristic (ROC) curves were calculated for seven GP thresholds and eight low-confidence genotype thresholds (Supplementary Fig. S1). Each curve followed a similar trajectory. However, at a specific GP threshold, low-confidence genotype thresholds lead to different outcomes for true-positive and false-positive rates (TPRs and FPRs, respectively). For example, at GP > 0.7, a low-confidence genotype threshold of two is required for a TPR > 0.8 and a FPR < 0.5 (Fig. 4a). By comparison, to meet those same criteria at a threshold of GP > 0.9, a low-confidence genotype threshold of four is required (Fig. 4b). These results indicate that at higher GP thresholds, a greater level of robustness is achieved when selecting a low-confidence genotype threshold, as small changes in threshold values do not lead to large changes in the number of variants removed.

False discovery rate (FDR), defined as the proportion of remaining genotypes that were incorrectly imputed, and the keep rate, which is the proportion of total genotypes that remain after filtering, were also analyzed for the thresholds defined above (Methods) (Supplementary Fig. S2). This was done to assess how much data was lost by filtering and to determine the number of imputation errors that remain within the dataset after filtering. Similar to results shown in ROC curves, a higher GP threshold provided a higher level of robustness for selecting a low-confidence genotype threshold. For example, at GP < 0.7, a low-confidence genotype threshold of two leads to a keep rate > 0.8 and an approximate FDR of 0.015 (Fig. 4c). To achieve a similar keep rate and FDR at GP < 0.9, a low-confidence genotype threshold of four is required (Fig. 4d). These results show that higher GP thresholds are more suitable for filtering sites based on the number of low-confidence genotypes. This strategy allows for fine tuning of filtered results without a loss in accuracy. Ultimately, while a higher number of low-confidence genotypes will remain in the analysis with higher GP thresholds, our calculations of imputation accuracy and FDR show that these low-confidence genotypes are tolerated. For the remainder of our analysis, we used a confidence filter of GP < 0.9 and a low-confidence genotype threshold of four, which roughly corresponds to a genotyping error rate of 5%.

### Minor allele frequency impacts imputation accuracy

Imputation often performs poorly for rare alleles as statistical support is lacking. To determine the impact of low MAFs on low-pass imputation in dogs, we analyzed imputation accuracies using imputation quality score (IQS), a statistic that controls for allele frequencies by taking chance agreement into account (Lin et al. 2010). In addition, population MAF estimates were sourced from two different datasets, the imputed genotypes and the Plassais et al. (2019) dataset. These two alternative calculations of MAFs were chosen for further analysis as each calculation is available for similar low-pass imputation analyses.

**Fig. 5:**
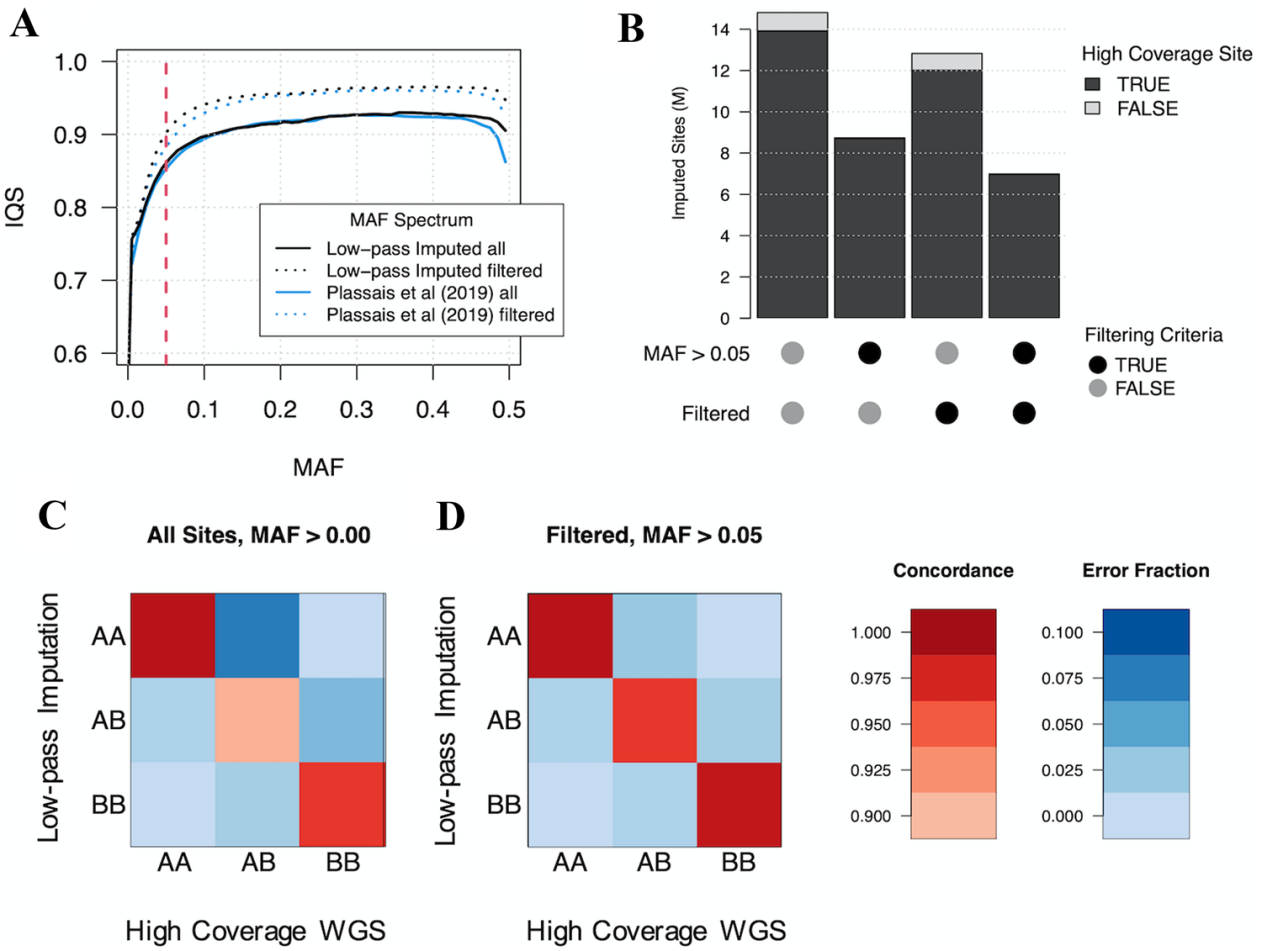
Imputation accuracy according to minor allele frequency and genotype. **a** Imputation accuracy according to imputed and Plassais et al. (2019) MAFs for all sites and quality-filtered sites. Imputation accuracy is measured as mean imputation quality score (IQS), an imputation accuracy statistic that accounts for the probability an allele is correctly imputed by chance. The red dotted line indicates a MAF of 0.05. **b** The number of sites remaining after filtering for MAF > 0.05 and for low-confidence genotypes < 5% as indicated by the “Filtered” label. Bar colors represent imputed sites that were either found or missing from the high-coverage WGS dataset. **c** Concordance and error rates for all genotypes, expressed as a fraction of the total number of high-coverage WGS genotypes. **d** Concordance and error rates for genotypes in sites with < 5% low-confidence genotypes and MAFs > 0.05. Rates are expressed as a fraction of the number of high-coverage WGS genotypes that meet the corresponding filtering criteria

Both sources of MAFs expressed similar IQSs, where the largest differences are due to whether imputed data were filtered. At MAFs > 0.1, the IQS of the unfiltered genotypes plateaued at approximately 0.91, whereas filtered genotypes plateaued at approximately 0.95 (Fig. 5a). For MAFs < 0.1, imputation accuracy rates were slightly higher when MAFs were calculated from imputed genotypes rather than from the Plassais et al. (2019) dataset. At a MAF of 0.05, only the filtered genotypes that were partitioned according to imputed MAFs had an IQS > 0.9, providing the most accurate results for low MAF genotypes.

We next analyzed the impact of MAF and filtering on imputation accuracy for different genotypes. Imputation accuracy was poorest for heterozygous genotypes, especially at low minor allele frequencies, indicating that heterozygous genotypes were least likely to be correctly imputed. Conversely, homozygous reference imputation was most accurate at lower MAFs, which is likely due to increased chance agreement between the high number of reference genotypes (Supplementary Fig. S3). In addition, non-reference concordance, mean *r*^2^, and IQSs were measured across chromosome 38 so that results can be compared to other analyses that use a different accuracy measurement (Supplementary Fig. S4). As expected, all three measures show higher levels of imputation accuracy at higher MAFs and a clear improvement for accuracy measurements for filtered genotypes. Together these results demonstrate that removing sites with a MAF < 5% retains a highly accurate set of genotypes, whereas filtering on GP values increases overall imputation accuracy. In addition, we show imputation errors are more likely for heterozygous sites, and MAF estimates derived from imputed genotypes are suitable for filtering by MAF.

An important consideration when filtering variants is the total number of sites remaining. Removing variants with a MAF < 0.05 and filtering out variants according to GP values result in a total of 7 M remaining sites. Most sites are removed due to the MAF cutoff rather than GP filtering. In addition, almost all sites remaining after filtering correspond to a site found in the high-coverage WGS dataset, highlighting the quality and accuracy of the final dataset (Fig. 5b). An additional impact from filtering is the reduced variability between genotype-specific imputation errors and concordance rates. For example, prior to filtering, the overall concordance rate for high-coverage WGS heterozygous genotypes with low-pass imputed genotypes was 90.8%, with 7.2% of those imputed as homozygous reference and 2.0% imputed as homozygous alternate (Fig. 5c). Conversely, after filtering, 96.4% of these genotypes were imputed correctly, with approximately 2.3% of these genotypes imputed as homozygous reference and 1.3% of genotypes imputed as homozygous alternate (Fig. 5d). Therefore, after filtering according to GPs and MAFs, overall concordance rates increase relative to unfiltered genotypes. In addition, imputation errors are more evenly spread across the other genotypes (Supplementary Table S6).

**Fig. 6:**
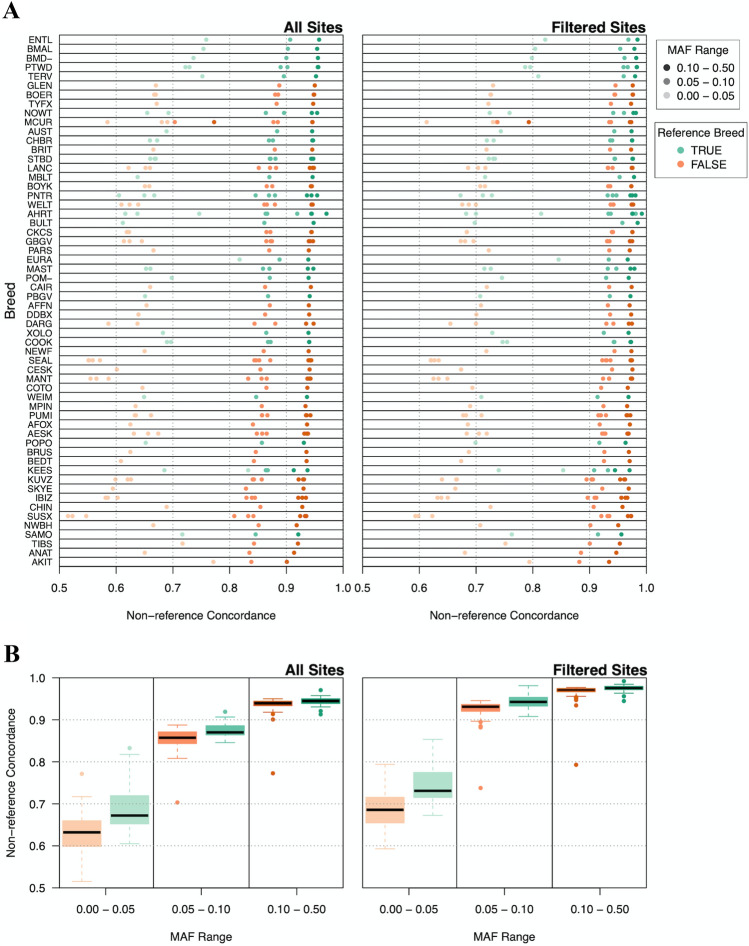
Imputation accuracy of dog breeds. **a** Individual dog breed imputation accuracy. Dog breeds are displayed on the Y axis with imputation accuracy on the X axis as non-reference concordance. Accuracy rates are displayed for all sites (left) and sites that remain after quality filtering (right). The shading of each data point indicates imputation accuracy of SNVs within a specific MAF range. Green data points indicate breeds present in the reference panel, while orange points indicate breeds absent from the reference panel. Breeds are ranked according to their median imputation accuracy for all sites. Imputation accuracies are displayed for each member of the breed. **b** Imputation accuracy of reference and non-reference breeds according to MAF

To determine whether the breed composition of the refence panel used for imputation impacts imputation accuracy, we measured non-reference concordance according to breed and MAF. Overall, breeds whose members showed the highest levels of imputation accuracy prior to filtering, such as the Entlebucher sennenhund, Belgian malinois, Bernese mountain dog, Portuguese water dog, and Belgian tervuren, also had members within the imputation reference panel (Fig. 6a) (Supplementary Table S7). Importantly, four of these five breeds were among the top 20 most highly represented breeds within the reference panel, with each containing at least nine members within the reference panel (Fig. 1c). Alternatively, breeds with the lowest levels of imputation accuracy usually lacked members in the reference panel (Fig. 6a). Moreover, breeds with low imputation accuracy that did have members in the reference panel, such as the Samoyed and Keeshond, had poorer representation than high imputation accuracy breeds, with both breeds containing only three members within the reference panel (Supplementary Table S2). Furthermore, imputation accuracy rates of reference panel breed members were consistently significantly higher than non-reference panel breed members across all MAF ranges (Welch Two Sample *t* test, *P* value < 0.05), where effect sizes were most pronounced at low MAFs (Fig. 6b) (Supplementary Table S8). These results indicate the importance of breed representation for improving imputation accuracy.

### Imputation errors reduce statistical power

**Fig. 7:**
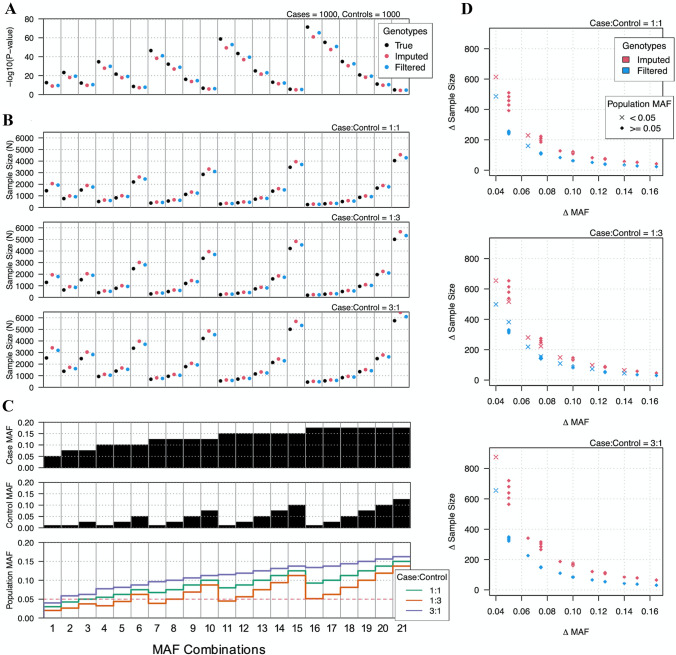
Impact of imputation errors on case–control GWAS. **a** Significance of case–control GWAS at multiple MAFs. True genotypes are represented by black circles, where the frequency of heterozygous and homozygous variants follow Hardy–Weinberg equilibrium. Red circles represent the outcomes of significance testing on imputed genotypes, while blue circles represent outcomes after filtering imputed genotypes. Note, decreases in significance were due to estimates of errors introduced during the process of imputation. Imputation errors were modeled according to the probability of a given genotype being imputed as any other genotype at any stated MAF. **b** Power analysis of significance testing for case–control GWAS of true and imputed genotypes. Y axis shows required samples size to reach a statistical power of 0.80. Each individual plot shows different case–control ratios. Power was calculated for a 2 × 2 chi-square test for significance level 5 × 10^–8^, where effect size was calculated as Cohen’s w. **c** Case and control MAFs used for each significance test analysis and the combined population allele frequency for each case and control configuration. Panels (**a**–**c**) are arranged in columns so that results presented in **a** and **b** correspond to the MAF configurations and values displayed in (**c**). **d** Additional samples required to reach sufficient power for imputed genotypes. Delta sample size is the difference between required sample sizes for true genotypes and imputed or quality-filtered imputed genotypes. Delta MAF is the difference in MAFs between cases and controls. Delta MAF is proportional to effect size

After characterizing genotyping errors introduced by imputation, we simulated the impact of these errors on case–control association tests to determine best practices for study designs involving low-pass imputed genotypes. Given a specific genotype detected by high-coverage WGS within a specific 0.01 population MAF interval, imputation errors were characterized as the probability of imputing either a homozygous reference, heterozygous, or homozygous alternate genotype. Overall, the imputation process led to decreased significance levels, suggesting that imputation errors may cause statistical significance to be lost for certain experimental configurations (Fig. 7a). To investigate the loss of statistical significance in the context of study design, we performed a power analysis with a focus on the number of samples required to reach sufficient power at 0.8 (Fig. 7b). Specifically, we tested 21 case and control population MAF combinations over three different case–control ratios and used the MAF of the entire population to simulate imputation errors (Fig. 7c). Case and control genotypes were based on Hardy–Weinberg equilibrium and were calculated using each population’s MAF (Methods). Results showed that experimental configurations for which the difference between case and control was smallest required the largest sample sizes. The required sample sizes were also higher for scenarios with non-1:1 case–control ratios, indicating that asymmetric population sizes increase the impact of imputation errors.

Importantly, the increased imputation accuracy afforded from quality-filtered data translated to reduced sample size requirements for achieving sufficient power (Fig. 7b). Also, the difference in MAFs between cases and controls was strongly predictive. MAF differences of 0.05 required > 500 additional samples for unfiltered imputed genotypes to achieve the same power as the true genotypes, while MAF differences of 0.1 required approximately 100 additional samples (Fig. 7d). Similar to the higher number of total samples required for asymmetric populations, sample requirements for imputed data were also increased. Finally, to achieve adequate power, quality-filtered imputed data require approximately half the number of additional samples as non-filtered imputed data (Fig. 7d). Together, these results indicate the importance of considering imputation error in the role of study design. Importantly, the impact from imputation errors is inversely proportional to the effect size of the association.

## Discussion

Genotype imputation is a valuable tool for determining missing genotypes and improving power to detect genome-wide associations. It also provides an opportunity to combine samples genotyped on different platforms into a single analysis (Hayward et al. 2019; Marchini et al. 2007; Uh et al. 2012). Ultimately, this increases the amount of available data by allowing datasets to be reused in larger studies (Ho and Lange 2010; Zhuang et al. 2010). However, imputation techniques have gone a step further and now facilitate genotyping of poor quality or low-pass sequencing data, which often lack sufficient coverage for genotyping software to assign confident calls. Imputation reference panels and algorithms provide the additional statistical support required to assign genotypes to individual samples (Rubinacci et al. 2021; Wasik et al. 2021). However, since the samples used to impute genotypes are different from those that undergo low-pass WGS, imputation has the potential to introduce genotyping errors and biases. Therefore, we investigated the impact of imputation on genotyping in dogs, an important genetic system whose unique population structure, defined by strict breeding programs over hundreds of years (Ostrander et al. 2017), may uniquely influence imputation accuracy. To characterize imputation errors and provide best practices for analyzing low-pass imputation data in dogs, we tested imputation for 97 high-coverage WGS dog samples by downsampling to approximately 1× coverage per sample. Imputation errors were detected by comparing the imputed genotypes to genotypes determined using high-coverage WGS data. We analyzed these errors in the context of genotype quality filtering, imputation error biases, the role of MAFs, and their impact on case–control analyses.

Our analysis of case–control association tests of imputed genotypes provides the necessary information to outline best practice guidelines for working with low-pass imputed genotypes. We show herein that the most important factor to consider is the expected allele frequencies in both cases and controls for any potentially associated markers. The most common error observed was imputing a heterozygous genotype as homozygous reference, leading to an overall reduction in observed effect size, thus requiring more samples to reach sufficient power. Importantly, these reduced associations were most pronounced when the allele frequency difference between cases and controls was small (≤ 0.05). Therefore, best practices in study design for low-pass imputation are to investigate genetic associations with medium-to-large effect sizes. Alternatively, if effect sizes are likely small, investigators need to consider increasing sample sizes, balance between case and control populations, and the role of quality filtering for improving overall accuracy.

Quality filtering imputed genotypes is achieved by removing sites that are above the genotype failure rate threshold of 5%, where failed low-confidence genotypes are those with maximum genotype probabilities (GP) < 0.9. These genotype failure rates and GP thresholds were chosen as they were well calibrated to optimally reduce imputation errors. We demonstrate that quality filtering can improve IQSs by a value of ~ 0.04 and reduce required increases in sample sizes for sufficient power by as much as 50%. Essentially, these improvements are achieved by removing ~ 20% of sites that together share a disproportionate number of the imputation errors. In addition, removing sites with MAFs < 0.05 removes those with comparatively lower imputation accuracies and sites that aren’t found in high-coverage WGS datasets.

After both quality and MAF filtering ~ 7 M SNVs remain, with SNVs found approximately every 360 bp. Despite removing the majority of SNV markers through filtering, sufficient numbers of SNVs remain for association analyses. Typically, association studies within breeds demarcate regions on the order of one Mb (Karlsson et al. 2013; Lindblad-Toh et al. 2005; Sutter et al. 2004; Vaysse et al. 2011), whereas across breeds, the scale of LD is approximately 10–100 kb (Karlsson et al. 2007; Parker et al. 2017). In addition, currently available canine DNA genotyping arrays contain just over 710,000 markers (Axiom™ Canine HD Array), one tenth the total number of markers available from low-pass imputation after quality and MAF filtering. Therefore, the benefit of increased genotyping accuracy using filtering likely exceeds the cost incurred from reduced marker density.

An additional consideration in performing GWASs using imputed data with small effect sizes is the required increase of both the case and control populations to reach sufficient power. For filtered genotypes with population MAF differences of 5%, approximately 250 additional samples with equal proportions of cases and controls are required to reach sufficient power. Importantly, as the MAF difference between cases and controls decreases, the number of additional samples required for imputation of low-pass WGS data to reach the same power as high-coverage WGS appears to grow exponentially. This is perhaps linked to increased rates of imputation errors at MAFs < 0.05.

Finally, for best practices study design, balance between case and control populations should also be addressed. The impact on power from unbalanced case and control populations is most prominent at low MAF differences between cases and controls. When that ratio favors either population, there is an overall loss of power. For example, at a case–control MAF difference of 5%, where the ratio of cases to controls is either 1:3 or 3:1, > 300 additional samples are required to reach sufficient power, whereas if the ratio is 1:1, only 250 additional samples would be required. This is because a lower number of total associated alleles, as opposed to proportion of alleles, within either the cases or controls, increases the likelihood that accumulation of imputation errors can cause a loss of statistical significance.

There are two strategies for developing reference panels. The first is to use a population with closely matched ancestry to that of the dataset under study, and the second is to use as many samples as possible with less consideration of ancestry. While not evaluated in the context of low-pass sequencing imputation, analysis of DNA arrays shows that reference panels matched to the population of interest outperform diverse reference panels of similar sizes (Bai et al. 2019; Mitt et al. 2017; Yoo et al. 2019; Zhou et al. 2017). This would suggest that larger refence panels are preferable if they contain sufficient representation of the study population. However, the addition of diverse samples to a reference panel can decrease imputation accuracy at low MAFs, where the magnitude of this effect varies according to the population being studied (Bai et al. 2019). These observations were for population-matched reference panels of ~ 100 samples; additional diverse samples increase the reference panels to over 860 samples (Bai et al. 2019). Other analyses compared references panels of > 1500 samples to the Haplotype Reference Consortium (HRC) reference panel (http://www.haplotype-reference-consortium.org/) (McCarthy et al. 2016; Mitt et al. 2017; Yoo et al. 2019; Zhou et al. 2017), which consists of 32,611 samples, indicating the potential for increased resolution in human studies compared to canine studies, which used a panel of just 676 samples from 91 breeds (Piras et al. 2020). Altogether, at MAFs > 0.05, human imputation studies conducted using DNA array genotypes show non-reference concordance rates > 97.5% and mean *r*^2^ values > 0.95 (Mitt et al. 2017; Yoo et al. 2019; Zhou et al. 2017). By comparison, prior to filtering, non-reference concordance rates for low-pass sequence imputation in dogs were at ~ 95% and had mean *r*^2^ values between 0.90 and 0.94 (Supplementary Fig. S4), highlighting the potential gains that can be achieved from improved canine reference panels.

An important initiative that may help address shortcomings in available high-coverage WGS canine samples is the Dog10K project which aims to achieve 10,000 modest to high coverage dog genomes representing an array of canine genetic diversity (Ostrander et al. 2019). Similar to the current dog reference panel, the initial phase of the Dog10K project prioritizes collecting samples from as many modern breeds as possible. This is important as dog domestication comprises of multiple population bottle necks, promoting the emergence of breed-specific genetic variation (Ostrander et al. 2017). Importantly, if certain breeds are absent from reference panels, their breed-specific genetic variation would remain hidden from imputation analyses. Therefore, care must be taken in deciding whether an optimal reference panel is being used to impute missing genetic variation in a given cohort.

Many canine mapping studies focus on traits that segregate across breeds, with the notion that breeds sharing recent common ancestry will likely share the same genetic underpinnings for any given trait (Parker et al. 2017). While multiple breeds are often included within a single analysis, creating many breed-specific reference panels is not feasible. As large haplotype blocks are shared between many breeds and breed clades, perhaps a large reference panel representing a greater number of breeds could provide even higher levels of imputation accuracy than a breed-specific reference panel. This idea is supported by an array-based imputation analysis that tested the imputation accuracy for a group of poodles with three different refence panel configurations. Results showed a composite panel of poodles and non-poodles outperformed the poodle only and the non-poodle reference panels (Friedenberg and Meurs 2016). A key finding from our analysis is that SNV discovery in individual dogs was similar between reference panel and non-reference panel breeds (Fig. 2c), and while imputation accuracy rates scored highest in reference panel breeds, several non-reference panel breeds outperformed most reference panel breeds (Fig. 6a). This was likely because many of the breeds reflected in the test samples belonged to breed clades represented in the reference panel. However, since the original VCF for the reference panel was unavailable, and breeds were identified from matching IDs across databases, breed membership for 122 dogs could not be determined. In addition, 98 samples in the reference panel were either free breeding village dogs or other canid species. In our test samples, 31 dogs from 14 breeds had not been previously associated with a clade (S3 Table). Five were terrier breeds that likely belong to the primary terrier clade and two were spaniel breeds that can be assigned to the spaniel clade. Since breeds of European origin are heavily represented in our refence panel, and most breeds with no clade assignment are of European ancestry, it is likely that non clade assigned test sample haplotypes are at least partially represented in the reference panel. Supporting this idea is the observation by Hayward et al. (2019) that high levels of imputation accuracy are achieved when imputing mixed breed dogs using sequence from pure breeds. As more high-coverage WGS samples become available through initiatives such as Dog10K, more optimal reference panel designs can be constructed.

One additional improvement in imputation accuracy may derive from the choice of imputation algorithm. Currently, available tools for imputation of low-pass WGS data include STITCH (Davies et al. 2016), Beagle (Browning and Browning 2016), GeneImp (Spiliopoulou et al. 2017), GLIMPSE (Rubinacci et al. 2021), and loimpute (Wasik et al. 2021). Our analyses used loimpute, as it had already been implemented with a dog reference panel and used by the canine genomics community (Piras et al. 2020). However, if low-pass sequencing imputation approaches in dogs are going to improve, other algorithms need to be appropriately assessed with accuracy across a range of MAFs. Currently, human studies demonstrate that GLIMPSE outperforms the other algorithms in terms of both accuracy and required computations resources (Rubinacci et al. 2021). The largest differences were observed for variants with MAFs < 1%. For common alleles, imputation accuracy was highest for GLIMPSE and Beegle, which was followed closely by loimpute and GeneImp.

Our work provides the first in-depth analysis of low-pass WGS and imputation in canine genomics, providing a road map for analysis in other non-human species. By comparing genotypes imputed from downsampled reads to a high-coverage truth set, we have rigorously investigated the nature of imputation errors and their biases. We were able to optimize filtering strategies to improve accuracy rates and demonstrate the impact imputation errors have on case–control GWAS. Our results inform a series of best practices guidelines and demonstrate the utility of this quickly evolving resource for future analyses. Altogether, widespread adoption of low-pass sequencing and imputation within the canine genomics field, together with investment in developing improved reference panels, will lead to more high-powered analyses and successful discovery of genotype–phenotype associations.

## Methods

### Sample selection

Test samples were selected on the basis of whether they belonged to known breeds, were absent from the reference panel, and had mean sequence coverage levels > 15×. At the time of the analysis, the test samples were not yet publicly available, guaranteeing their absence from the Gencove Inc. reference panel, thus providing an unbiased test of imputation performance. To help identify samples used in the reference panel, Gencove, Inc. provided a list of sample IDs. Most of these IDs were matched to known samples within the Plassais et al. (2019) dataset, which was used to estimate likely variant population frequencies within the Gencove reference panel. Breed names were based on annotated records and clade membership was based on previously published results (Parker et al. 2017). Breeds with no recorded clade membership were assigned to a clade based on their phenotype, historical data, or phylogenetic clustering in Plassais et al. (2019).

### Variant calling and imputation

Sample reads were mapped to CanFam3.1 using BWA-mem (Li 2013). Variant calling was performed using GATK4 best practices (McKenna et al. 2010). Base quality score recalibration and duplicate marking were applied to each sample (DePristo et al. 2011; Van der Auwera et al. 2013), and haplotypecaller was used for variant discovery (Poplin et al. 2017). Average coverage was estimated using Samtools depth tool (Li et al. 2009). To simulate low-pass sequencing, BAM files were downsampled to approximately 1× coverage using the DownsampleSam tool from GATK4. To obtain the correct coverage level, the parameter “-p” was set as the sample’s mean coverage divided by one. Downsampled BAMs were converted to fastq files using samtools “fastq” function and were uploaded to Gencove, Inc. using the Gencove command line interface (CLI). Imputation was performed using loimpute as part of Gencove’s imputation pipeline with the “Dog low-pass v2.0” configuration (Piras et al. 2020; Wasik et al. 2021). Imputed genotypes were received from Gencove as a VCF for each individual. Individual VCFs were split according to chromosome. Each sample’s genotypes and genotyping statistics were merged to create a single dataset for each chromosome that contained all individuals. This task was performed using the program extract_genotype_wg.R which was written in R and used the vcfR package (Knaus and Grunwald 2017; Team 2013).

### Assessing imputation accuracy

Imputation accuracy was assessed by comparing imputed genotypes to high-coverage WGS genotypes. This was made possible by identifying sites shared across both datasets. Sites were considered shared if position, reference allele, and alternate allele were identical. Importantly, all multiallelic sites in all VCFs were split into biallelic states using the bcftools “norm” function with the “-m –” parameter to ensure that all potential allelic combinations were matched (Li 2011). Sites where all samples were homozygous for the reference allele were removed from the analysis. A genotype for a given individual at a particular site was considered concordant if the imputed genotype was identical to the genotype determined using high-coverage WGS. Imputation errors were those that were not identical between the high-coverage WGS dataset and imputed dataset for a given individual at a given site. The total number of concordant genotypes per sample was calculated using the program “venn_filter_wg.R”. Once filtering thresholds were determined (below), imputation accuracies were determined according to MAF intervals of 0.01 using the program “gt_by_af.R”. IQS was calculated by following the methods described in Lin et al. (2010). Mean R2 values were determined by calculating the squared Pearson correlation coefficient of genotype dosages between WGS and imputed genotypes across all samples within a given MAF range.

### Determining filtering thresholds

The imputation process provides genotype probabilities as a measure of confidence regarding whether a call is homozygous reference, heterozygous, or homozygous alternate. The max genotype probability (GP) is the level of confidence for the imputed genotype. We therefore investigated the relationship between GP and the proportion of concordant genotypes between our imputed and high-coverage WGS datasets to determine optimal strategies for filtering imputed variants. Low-confidence or failed genotypes were identified according to their GP values and variant sites were filtered out if the number of low-confidence genotypes was above a given threshold. (Fig. 3a). Filtering strategies were evaluated according to the number of remaining genotypes after filtering and the proportion of these genotypes that were concordant with the truth set. These values were further investigated in terms of the true-positive rate (TPR), false-positive rate (FPR), false discovery rate (FDR), and the number of genotypes kept after filtering (Fig. 3b).

### Simulation of imputation errors

Imputation errors were simulated using probabilities derived from the observed fraction of any given genotype that was incorrectly imputed. Error probabilities were also grouped according to population MAFs. For example, for sites with MAFs between 0.03 and 0.04, 5% of heterozygous sites may be imputed as homozygous reference, whereas for sites with MAFs between 0.10 and 0.11, 3% of heterozygous sites may be imputed as homozygous reference. This strategy is used to capture the MAF impact on imputation accuracy and error rates. The imputed genotypes, $I$, were stored as a 1 × 3 matrix, consisting of the imputed genotype counts for homozygous reference, heterozygous, and homozygous alternate genotypes. The values for $I$ were calculated as $I=GP$, where $G$ is the counts for the true starting genotypes stored in a 1 × 3 matrix and $P$ is a 3 × 3 matrix of the probabilities a true genotype is imputed as any other genotype. In $P$, rows represent the true starting genotype and columns represent the imputed genotypes. Importantly, the values used for $P$ depend on the population MAF and whether the genotypes were filtered for quality. A total of 100 $P$ matrices were defined, with one matrix for each 0.01 MAF interval between 0 and 0.5 for both quality-filtered and unfiltered imputation error rates (Supplementary Fig. S5).

### Association analyses and statistical power calculations

Case–control association analyses were performed as a chi-square test on a 2 × 2 contingency table, measuring the association between cases and the presence of the minor allele. Association tests were carried out on the true genotypes, imputed genotypes, and quality-filtered imputed genotypes. Power was calculated using the “pwr” package in R (Champely et al. 2020), which uses Cohen’s $w$ to calculate effect size. Probabilities for the null hypothesis were calculated as if the minor allele was evenly distributed across both cases and controls. Significance levels were set at 5 × 10^–8^ and power was calculated across a variety of case and control MAFs for all population sizes between 100 and 7000. Required sample sizes for sufficiently powered analyses were identified as the lowest sample size that could achieve a power level of 0.8 or greater for a particular case–control analysis.

## Supplementary Information

Below is the link to the electronic supplementary material.

Supplementary file1 (DOCX 503 kb)

Supplementary file2 Table S1 Sample-specific metadata. Table S2 Breed composition of datasets. Table S3 Breed clade membership. Table S4 Breeds not included in previous phylogenetic analyses and have no clade membership. Table S5 Mean chromosome 38 coverage levels at variant sites. Table S6 Individual concordance rates between high-coverage WGS and low-pass imputed genotypes on chromosome 38. Table S7 Imputation accuracy rates for individual samples measured as non-reference concordance. Table S8 Welch Two Sample t-test for imputation accuracies of reference panel breed samples and non-reference panel breed samples across allele frequency ranges (XLSX 143 kb)

## Data Availability

Test samples used in this analysis are deposited under the BioProject accession PRJNA648123 and PRJNA726547. Individual accessions for each sample are recorded in S1 Table.

## Code availability

All original software used here can be obtained from the following URL: https://github.com/NHGRI-dog-genome
