# Supplementary material for: Best practices for analyzing imputed genotypes from low-pass sequencing in dogs
Source: Mamm Genome. 2021 Sep 8;33(1):213–29. doi: 10.1007/s00335-021-09914-z (PMC8913487; doi:10.1007/s00335-021-09914-z)
Supplement: Supplementary file 1 — Supplementary file1 (DOCX 503 kb) [file 335_2021_9914_MOESM1_ESM.docx]

**Best practices for analyzing imputed genotypes from low-pass sequencing in dogs**

Reuben M. Buckley, Alex C. Harris, Guo-Dong Wang, D. Thad Whitaker, Ya-Ping Zhang, and Elaine A. Ostrander

Supplementary Figures

**Supplementary Fig. S1: Receiver operator characteristic (ROC) curves at different GP thresholds.** Genotypes below GP thresholds shown in the lower right corner of each plot are designated as low confidence. Numbers above each point represent low confidence rate thresholds for removing sites. Sites with a total number of low confidence genotypes greater than or equal to the threshold are filtered out. Grey dashed lines represent ROC curves for other GP confidence threshold values.

**Supplementary Fig. S2: The proportion of variants remaining after filtering at different GP thresholds and the corresponding FDR.** Again, as in Supplemental Figure S2, genotypes below GP thresholds shown in the lower right corner of each plot are designated as low confidence. The numbers above each point represent low confidence rate threshold values and grey dashed lines represent curves for other GP confidence thresholds.

**Supplementary Fig. S3: Imputation accuracy for each individual allele according to various MAF spectra**. Imputation accuracy is expressed as the proportion of high coverage WGS genotypes concordant with low-pass imputed genotypes.

**Supplementary Fig. S4: Comparison of various imputation accuracy measurements across chromosome 38. Accuracy measurements including non-reference concordance, mean r2, and IQS. Whole genome measurements are included for IQS to show chromosome 38 is representative of the whole genome.**

**Supplementary Fig. S5: Simulating imputation errors.** 1) Definitions of terms used throughout figure. Numbers 0 to 2 represent the number of individuals for each genotype in the 1x3 true genotype matrix G and the 1x3 Imputation matrix. N represents the total number of individuals in the analysis. This number is equal for true genotypes and imputed genotypes. 2) The 3x3 matrix which shows the imputation probabilities for each of the True genotypes. 3) The equation to calculate the number of individuals for each genotype in the imputation matrix. For clarity, the calculations for each genotype have been expanded. 4) Schematic illustrating the changing imputation probabilities for P matrices calculated across different MAF ranges.
